# Sporadic Creutzfeldt-Jakob Disease Causing a 2-Years Slowly Progressive Isolated Dementia

**DOI:** 10.3233/BEN-2009-0238

**Published:** 2009-12-07

**Authors:** Álvaro Machado, Manuel Ribeiro, Margarida Rodrigues, Carla Ferreira, Inês Baldeiras, M. Helena Ribeiro, Isabel Santana, Rui Almeida, Lígia Castro, Stirling Carpenter

**Affiliations:** ^1^Neurology DepartmentHospital de São MarcosBragaPortugal; ^2^Neuroradiology DepartmentHospital de São MarcosBragaPortugal; ^3^Neurochemistry LaboratoryHospitais da Universidade de CoimbraCoimbraPortugal; ^4^Neurology DepartmentHospitais da Universidade de CoimbraCoimbraPortugal; ^5^Neurosurgery DepartmentHospital de São MarcosBragaPortugal; ^6^Pathology DepartmentHospital de São JoãoPortoPortugal

**Keywords:** Creutzfeldt-Jacob disease, DWI, MV2, MRI, MRS, SPECT

## Abstract

A 47-year-old woman was seen for progressive behavioural and cognitive disturbances slowly evolving over a 1-year period. Neuropsychological evaluation disclosed moderate to severe impairment of all cortical functions. Besides this no other clinical abnormality was found. MRI diffusion weighted imaging disclosed hyperintense cortical lesions in a ribbon-like fashion, with restricted diffusivity. EEG showed no periodic sharp waves and CSF examination was normal, including protein 14.3.3. She was heterozygote on codon 129. Her cognitive function continued to decline and she was readmitted for further investigation at the 24th month of disease. Again no ataxia or involuntary movements were observed. MRI disclosed widespread hyperintense lesions over the entire cortex and, for the first time, also caudato-putaminal hyperintensity in T2-weighted images. EEG again failed to show periodic activity. Stereotactic biopsy disclosed moderate spongiform changes, astrocytosis and perivacuolar staining with prion-directed antibodies. Western blot analysis revealed prion type 2 mobility pattern. We discuss the clinical significance of this case: as dementia was the sole finding, and this was slowly-evolving over a 2-year period, MRI findings were the key factor suggesting a prion disease in a woman that otherwise would probably be diagnosed with a primary degenerative dementia.

## 1. Introduction

Prion diseases are a group of transmissible disorders that affect humans and several other mammalian species. They are believed to be caused by a proteinaceous infectious particle, resistant to most sterilization procedures, which is known as prion [11]. The prion appears to consist entirely of an abnormal isoform of a host encoded, membrane-associated, copper-binding protein of unknown function, called the prion protein (PrP) [7]. The normal PrP has high alpha-helix content and is soluble and protease sensitive.

In contrast, the prion has high beta-sheet content that makes it protease resistant, insoluble, and amiloidogenic [7]. The prion will induce the conversion of normal PrP into prion form. This gives the prion its infectious potential [7].

Creutzfeldt-Jakob disease (CJD) is the most common human prion disease, occurring in sporadic, familial, variant, and iatrogenic forms. Of these the sporadic form (sCJD) is by far the most common, with a stable worldwide prevalence of 1 per million per year [7]. The most widely accepted theory of prion diseases pathogenesis is a spontaneous misfolding of endogenous PrP into the prion isoform, in contrast to the exogenous pri-

ISSN 0953-4180/09/$17.00 © 2009 – IOS Press and the authors. All rights reserved on exposure that occurs in the variant and iatrogenic forms [7].

Clinical findings in typical sCJD are rapidly progressive dementia along with ataxia, visual disturbance, and myoclonus. Supporting features are periodic sharp wave complexes (PSWC) in the routine EEG, presence of 14.3.3 protein in the CSF, absence of mutations in the PrP gene, and basal ganglia and thalamus hyperintensities in long TR sequences. Usual histological features are the presence of spongiform change in the grey matter of cortex and basal ganglia, astrocytic proliferation, and variable neuronal loss.

There are two major phenotypic variants of classic sCJD, known as the Heidenhain and the Oppenheimer-Brownell variants. The former has a distinct clinical onset of visual disturbances, ranging from cortical blindness to several oculomotor abnormalities, and a rather rapid evolution to death [4]. The later is characterized by the onset of ataxia in the absence of other clinical features of sCJD, and by lack of both periodic sharp-wave complexes and basal ganglia hyperintensities [4].

The phenotype of CJD can be modulated, if not determined, by two variables: a naturally occurring polymorphism at codon 129 of the PrP gene giving MM, VV, or MV, and the pattern of electrophoretic PrP mobility (1 or 2) [9]. In combination, these features define six major subtypes of sCJD [9].

MRI has an increasing role in the diagnosis of human prion diseases, with typical appearances in both sporadic and variant forms [14]. This has been incorporated into the WHO case definitions for the variant form [14].

We present a unique case of a biopsy-proven MV2 sCJD patient that presented with isolated dementia and in which the final diagnosis was only suspected in face of the MRI findings.

## 2. Case history

A 47-year-old previously healthy female dressmaker was referred for cognitive decline. The first symptoms had been noted a year before. The progressive symptoms included difficulty in maintaining a conversation, forgetting whatever she was told, sewing buttons onto wrong places, ceasing to clean her house, confusion about money and responsibilities, and accumulation of debts. These changes appeared gradually over a year until she was invited to abandon her job.

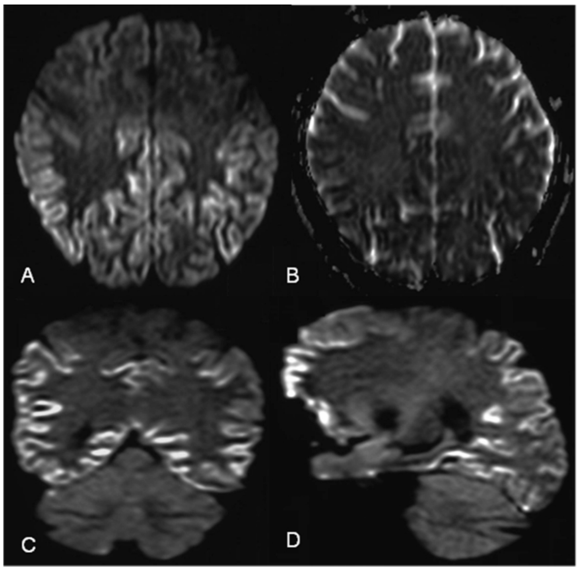

Fig. 1. A. Axial DWI shows a ribbon-like hyperintensity along the cortical gyri; B. ADC map shows correspondent low signal in the same location, consistent with restricted diffusivity of water molecules; Coronal DWI (C) and Sagital DWI (D) further demonstrate the aforementioned hyperintense gyriform lesions, involving temporal, parietal, frontal and occipital cortices.

At examination, besides cognitive impairment, no clinical abnormality could be detected. The MMSE score was 16. Comprehensive neuropsychological assessment disclosed severe executive dysfunction, moderate language disturbance, severe impairment of verbal, visual, semantic and autobiographical memory, and of visuoconstructive ability.

Brain MRI showed a linear high signal in temporal and parietal cortices, visible in FLAIR and especially in DWI sequences (Fig. 1A, C-D). In the ADC map a low signal was found, indicating restricted diffusion of water molecules (Fig. 1B). No abnormal signal was encountered in the basal ganglia or thalami. There was no sign of atrophy. EEG showed slow and irregular background activity. PSWC were not found. CSF was normal; protein 14.3.3 was absent; tau protein was 959.4 pg/mL (bellow the generally accepted cut-off value of 1350 pg/ml for sCJD) with phosphorylated tau (p-tau181) being 46.3 pg/mL. SPECT showed bilateral, though right predominant, temporoparietal perfusion defects, suggesting AD. Molecular genetics showed that codon 129 of the PrP was MV. PRNP was sequenced and showed no mutation. Extensive laboratory work-up was normal. Screening for neoplasm was negative. She was readmitted one year later, at the 24th month of disease. She did not have developed any other symptom or sign besides dementia, at that time with

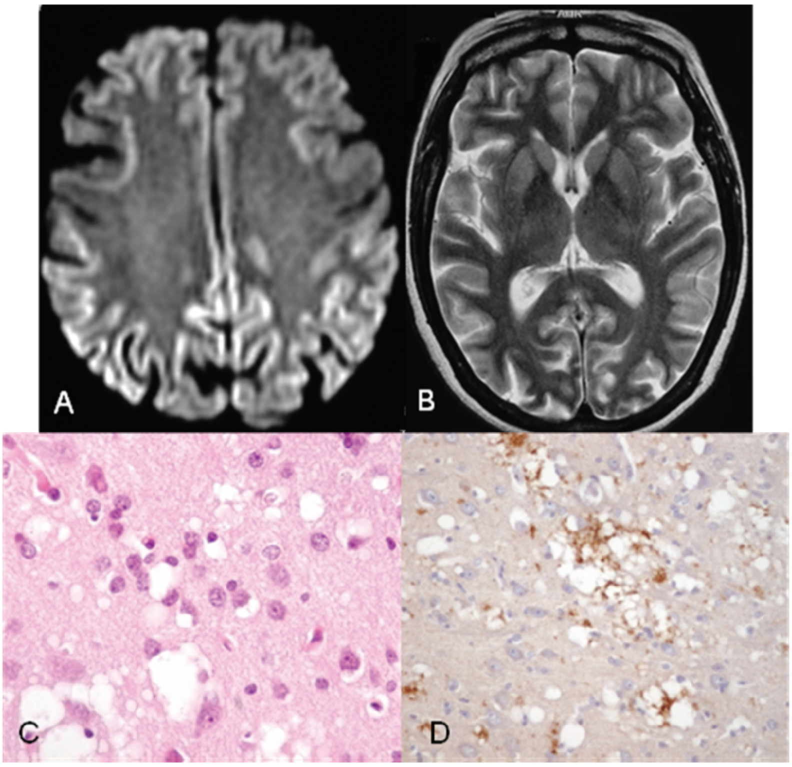

Fig. 2. A. Axial T2 shows symmetrical high signal lesions in caudate nuclei and putamina; B. Axial DWI shows widespread cortical hyperintensity; C. Hematoxilin-eosin preparation shows spongiform changes with astrocytic proliferation; D. Immunocytochemistry with antibody L42 reveals a perivacuolar staining pattern.

a MMSE score of 4. Brain MRI (Fig. 2A-B) showed not only extension of the cortical hyperintensities but also, for the first time, caudato-putaminal ones. Brain biopsy was decided on consultation with the family as the only way of excluding a potentially treatable disorder and also for prognostic information. Microscopic examination of the biopsy (Fig. 2C) showed moderately intense spongiform changes involving all laminae of the cerebral cortex, with the partial exception of the molecular layer. Neuronal loss was slight. No ballooned neurons were seen. Astrocytic nuclei were clearly increased in number, while the astrocytic cytoplasm was almost never enlarged. The white matter appeared normal. Immunocytochemistry with antibodies 3F4 and L42 showed the pattern described as perivacuolar staining (Fig. 2D). No plaque-like structures were seen. Western blot of brain tissue after treatment with protease K showed a type 2 pattern.

Presently, 40 months after disease onset, she is completely dependent and bedridden. In any time there was ataxia, and occasional myoclonic jerks were only detected in the last 8 months. The family is not willing to consent for autopsy.

## 3. Discussion

In the 6-fold classification of sCJD cases according to codon 129 of the PrP gene and the electophoretic type of the protease resistant prion, the present patient falls under the MV2 form, which is said to account for about 9% of sCJD cases [2,9]. Pathologically it is characterized not only by widespread spongiform changes, but also by amyloid plaques in the cerebellum. The immunological pattern is usually that of plaque-like deposits, even though amyloid plaques themselves are rarely visible in the cerebral cortex. The present case showed a perivacuolar pattern, which may coexist with a plaque-like pattern. The most commom forms of sCJD, those which give the classical picture of myoclonic rapidly evolving dementia with PSWC and high CSF 14.3.3 protein, are MV1 and MM1 [9]. Their immunological pattern of staining, which is diffuse and dot-like, is usually called synaptic. Compared with them, the MV2 form has a longer average duration of illness (18 months compared to 3.9 months), only rarely typical EEG changes or CSF changes, and a higher frequency of cognitive impairment at onset [2].

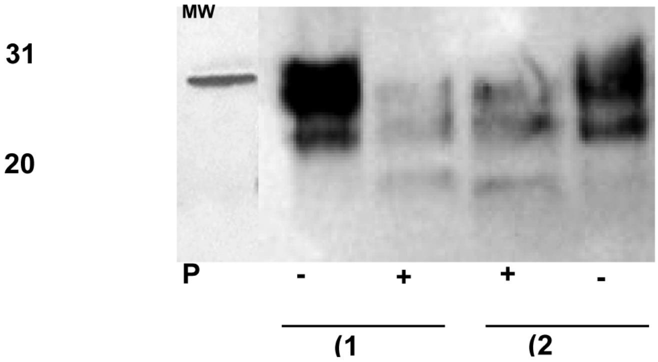

Fig. 3. Western blot analysis showing, in the left column, molecular 31 and 20 Kda markers (MW), a cerebellum extract from a sCJD PrP-type 1 case (1) and the brain biopsy from our patient (2), both without (−) and with (+) proteinase-K (PK) treatment.

MV2 patients may have a prolonged course, and about 18% of them may be monosymptomatic for more than 6 months [2]. In despite of this, we do not know of any other reported patient with exclusive cognitive decline over more than 24 months. Except for MRI findings, no other laboratory feature suggestive of sCJD could be found.

Since at present definite diagnosis of sCJD can only be obtained by pathological examination at biopsy or necropsy, there is a clear need for non-invasive diagnostic techniques. CSF 14.3.3 and EEG were previously considered the best auxiliary procedures, but in recent years MRI has been proposed as a superior diagnostic tool [3,6,8,14].

This is especially true for certain sCJD subtypes, such as MV2, where other investigations are frequently unhelpful, and MRI abnormalities are reported to be more striking [2].

Two patterns have been found to be highly predictive of sCJD: a high signal in the striatum and in at least 2 cortical gyri in a ribbon-like fashion, or else extensive cortical linear ribboning [6]. Using FLAIR and DWI sequences, sensitivity and specificity have been found to be over 90% [3,6,14]. This suggests that MRI, in particular if a thorough protocol is used, is a fundamental tool in sCJD diagnosis during life [14].

The high signal seen in DWI sequences has been interpreted as indicating restricted movement of water molecules, since corresponding low ADC values have been widely reported. On this basis various mechanisms have been proposed for diffusion restriction: (1) microvacuolation of neuritic processes, reflecting the characteristic spongiform changes [14], or (2) an abnormal hydrophobic isoform of the prion. Either of these mechanisms could explain the high DWI signal without corresponding T2-weighted hyperintensity [10]. When T2-weighted images also show a high signal, it is believed that the pathological substrate is astrocytic gliosis [13]. Subsequent spongiform changes could cause a decrease in the T2 signal towards an iso-intense pattern. The pathological substrate of concomitant high T2 and DWI signals remains uncertain.

The ribbon-like appearance of DWI abnormalities has also been encountered in a few other conditions than sCJD, such as herpes simplex meningoencephalitis, post-contusion states, and post-ictal state [5,8]. No problem should exist in clinically distinguishing these conditions from sCJD.

We have decided to perform the biopsy in the region most functionally affected as revealed by the neuropsychological assessment, since we were convinced that we would find disease-defining abnormalities, while minimizing post-surgical cognitive additional dysfunction. In the differential diagnosis we had to consider other entities, aside from the common causes of dementia such as Alzheimer's disease and frontotemporal dementia.

Because the case was clinically so atypical for sCJD, we also considered the possibility that this was a case of vCJD in a MV patient, as one person who died without brain involvement 5 years after a transfusion from a vCJD patient, and was heterozygote at codon 129, has been found to have immunoreactive prions in his lymphatic system [1]. If MV people do get sick with vCJD, no one knows what their clinical picture will be.

This report brings up several important points. 1) MRI is useful in raising the possibility of sCJD in atypical or unusually slow dementia, and it should be systematically done and include FLAIR and DWI sequences; 2) Studies addressing the role of MRI in sCJD should include cortical hyperintensities in a ribbonlike fashion as a diagnostic pattern; 3) This patient would have probably ended up with a diagnosis of either Alzheimer's disease or frontotemporal dementia if DWI had not been undertaken; 4) Neuropsychological assessment may be more useful than MRI or SPECT findings in choosing the biopsy site; 6) One must be aware of the phenotypical variation among sCJD subtypes to avoid missing cases.
